# Evolution and Adaptation in *Pseudomonas aeruginosa* Biofilms Driven by Mismatch Repair System-Deficient Mutators

**DOI:** 10.1371/journal.pone.0027842

**Published:** 2011-11-17

**Authors:** Adela M. Luján, María D. Maciá, Liang Yang, Søren Molin, Antonio Oliver, Andrea M. Smania

**Affiliations:** 1 Centro de Investigaciones en Química Biológica de Córdoba (CIQUIBIC), CONICET, Departamento de Química Biológica, Facultad de Ciencias Químicas, Universidad Nacional de Córdoba, Córdoba, Argentina; 2 Servicio de Microbiología and Unidad de Investigación, Hospital Son Espases, Instituto Universitario de Investigación en Ciencias de la Salud (IUNICS), Palma de Mallorca, Spain; 3 Department of Systems Biology, Center for Systems Microbiology, Technical University of Denmark, Lyngby, Denmark; Baylor College of Medicine, United States of America

## Abstract

*Pseudomonas aeruginosa* is an important opportunistic pathogen causing chronic airway infections, especially in cystic fibrosis (CF) patients. The majority of the CF patients acquire *P. aeruginosa* during early childhood, and most of them develop chronic infections resulting in severe lung disease, which are rarely eradicated despite intensive antibiotic therapy. Current knowledge indicates that three major adaptive strategies, biofilm development, phenotypic diversification, and mutator phenotypes [driven by a defective mismatch repair system (MRS)], play important roles in *P. aeruginosa* chronic infections, but the relationship between these strategies is still poorly understood. We have used the flow-cell biofilm model system to investigate the impact of the *mutS* associated mutator phenotype on development, dynamics, diversification and adaptation of *P. aeruginosa* biofilms. Through competition experiments we demonstrate for the first time that *P. aeruginosa* MRS-deficient mutators had enhanced adaptability over wild-type strains when grown in structured biofilms but not as planktonic cells. This advantage was associated with enhanced micro-colony development and increased rates of phenotypic diversification, evidenced by biofilm architecture features and by a wider range and proportion of morphotypic colony variants, respectively. Additionally, morphotypic variants generated in mutator biofilms showed increased competitiveness, providing further evidence for mutator-driven adaptive evolution in the biofilm mode of growth. This work helps to understand the basis for the specific high proportion and role of mutators in chronic infections, where *P. aeruginosa* develops in biofilm communities.

## Introduction

Mutation rate is controlled by several genetic systems. Particularly relevant is the mismatch repair system (MRS), which monitors the fidelity of DNA replication and recombination by repairing DNA polymerase errors and blocking recombination events between divergent (homeologous) DNA sequences [1], [2]. Consequently, defects in the MRS cause enhanced mutation rates and increases in recombination of divergent sequences, resulting in a phenotype known as the hypermutator or just mutator phenotype [1], [2]. Since mutation is the substrate for natural selection, the potential role of mutator phenotypes in adaptive evolution has attracted a huge biological and medical interest for decades. Indeed, early *in vitro* experiments, using *Escherichia coli* as model organism, indicated that the occurrence of mutators in bacterial populations provide a higher probability of generating advantageous mutations, allowing faster adaptation to new and/or changing environments [3].

In recent years, *Pseudomonas aeruginosa* has been recognized as an interesting model organism for investigations of the role of MRS in bacterial adaptive strategies [4]–[13]. *P. aeruginosa* is an important opportunistic pathogen causing chronic airway infections, especially in cystic fibrosis (CF) patients, but also in patients suffering from other chronic lung pathologies such as chronic obstructive pulmonary disease (COPD) or bronchiectasis [14]–[16]. The majority of CF patients acquire *P. aeruginosa* during early childhood, and most of them eventually develop a chronic infection, which is rarely eradicated despite intensive antibiotic therapy [14]. A striking feature of *P. aeruginosa* chronic infections is the very high prevalence (30–60%) of mutator strains [4], [17]–[20], mainly due to alterations in the *mutS* or *mutL* genes, the main components of the MRS [4], [5]. In contrast, detection of mutators is very infrequent in *P. aeruginosa* acute infections [21]. Moreover, recent research suggests that mutators may contribute to *P. aeruginosa* adaptation to the CF airways environment, since their presence is found to be linked to the acquisition of beneficial mutations [10], [22] such as those conferring antibiotic resistance [4], [5], [17], [20], [23], [24]. Nevertheless, the connection between mutator phenotypes and other adaptive strategies involved in *P. aeruginosa* chronic infections is still not clarified. *P. aeruginosa* most often resides within the thick CF mucus as biofilm structures [25], [26] that consist of organized consortia of bacteria embedded in a self-produced polymer matrix consisting of polysaccharide, protein and DNA. Importantly, it has been shown that the biofilm lifestyle provides *P. aeruginosa* with higher tolerance to antibiotics as well as resistance to phagocytosis and other components of the body's defense system [25], [26]. *P. aeruginosa* undergoes genetic diversification in the CF airways based on mutagenic events, leading to the emergence, selection and fixation of multiple phenotypic variants particularly adapted to specific anatomic niches in the CF lung [10], [22], [27]. Among them, mucoid, quorum-sensing deficient and smooth or rough small colony variants are the most common, and their emergence has been correlated with decreased lung function and poor prognosis for the patient [28]–[30].

Flow-cell biofilms formed by *P. aeruginosa* have been studied intensely in recent years [31], [32]. From these studies, it has been documented that biofilm formation is a complex multifactorial process regulated by both genetic and environmental factors [33]. Recognized stages in biofilm formation include initial bacterial attachment, micro-colony development, maturation of micro-colonies into large 3D-structures, and dispersion of bacterial cells from biofilms. Since mature micro-colonies constitute heterogeneous micro-environments, mainly defined by oxygen and nutrient limitations [34], a biofilm is a spatially structured environment [35], and adaptation to this environment may involve genetic diversification [36]. In fact, previous works reveals that for a wide range of bacterial species, biofilms grown *in vitro* generate extensive genetic diversity leading to the emergence of different biofilm adapted phenotypes [37]–[42]. Particularly, from *P. aeruginosa* biofilms, a wide range of colony morphology variants have been observed including small [43]–[46], rugous [44], [46], [47] and mucoid colony variants [8], [48], suggesting the existence of strong and varied selective pressures driving fixation of different phenotypes, similar to the diversification process observed in chronic CF airway infections [22], [27], [49]. Furthermore, recent evidence suggests that mutation may play a role in biofilm development [50] and that biofilm-mediated diversity in *P. aeruginosa* is based on intrinsically increased mutagenesis caused by endogenous oxidative stress [51], [52].

The parallel findings from CF airway samples and *in vitro* biofilms suggest that three adaptive strategies may be important for the development of *P. aeruginosa* chronic CF lung infections: (i) biofilm development, (ii) phenotypic diversification, and (iii) hypermutability. In order to understand better the connection between these processes we used the flow-cell biofilm model system to investigate the impact of the *mutS* mutator phenotype on development, structure, dynamics, diversification and adaptation of *P. aeruginosa* biofilms.

## Results

### Structural dynamics of biofilms formed by wild-type and mutator strains of P. aeruginosa

Biofilm development of two *P. aeruginosa* strains, the reference strain PAO1 [53] and the environmental strain Hex1T [54] as well as their *mutS* isogenic mutants, PAOMS [55] and Hex1TMS [6], respectively, was monitored in flow-cell systems and characterized by scanning laser confocal microscopy (CLSM). Cellular adhesion was examined for all strains by assessing the number of cells adhering to the flow-cell glass cover slides after medium flow was resumed. Both Hex1TMS and PAOMS *mutS* strains showed comparable adherence [3.7±0.2×10^4^ cells/cm^2^ and 3.6±2×10^4^ cells/cm^2^ (mean ± SD), respectively] to the Hex1T and PAO1 wild-type strains [3.9±1.3×10^4^ cells/cm^2^ and 5.3±2.4×10^4^ cells/cm^2^ (mean ± SD), respectively], suggesting that a high mutation rate does not affect the initial attachment of cells, at least under the conditions assayed here.

**Figure 1:**
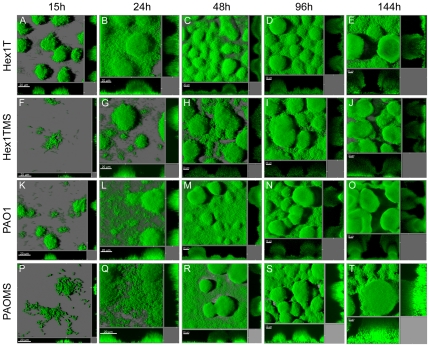
Biofilm formation by *P. aeruginosa* wild-type and mutator strains. The images shown are 3D-reconstructed images of biofilm dynamics of Hex1T (A-E), Hex1TMS (F-J), PAO1 (K-O) and PAO1MS (P-T). The development of mutator biofilms was exhaustively studied in parallel to wild-type biofilms. All strains were GFP-tagged and grown in continuous flow system in FAB medium supplemented with 0.3 mM glucose for 144 h. Confocal images were acquired following 15, 24, 48, 96 and 144 h of growth.

Biofilm development was investigated by acquiring CLSM micrographs at 15, 24, 48, 96 and 144 h and subjecting the digital three-dimensional images to COMSTAT image analysis. At 15 h after inoculation, the two wild-type strains formed small, ball-shaped micro-colonies (Figure 1 A and K). In contrast, the respective mutator strains showed significantly less organized micro-colonies, in which singles cells could still be distinguished (Figure 1 F and P). These differences were also documented from the quantitative COMSTAT analysis, showing that at 15 h the mutator strains displayed significantly lower biomass accumulation and thickness but higher surface to volume ratios (Figure S1). After this lag in biomass development both wild-type and mutator strains showed a normal maturation pattern with micro-colony differentiation leading to development of mushroom-shaped multicellular structures reaching full maturation on day 6 (Figure 1).

**Figure 2:**
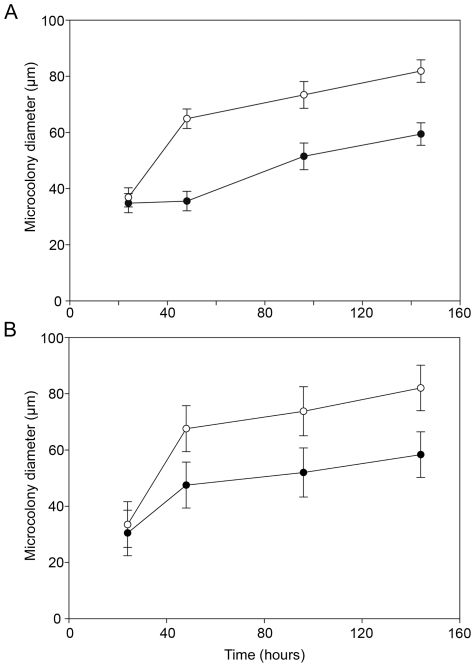
*P. aeruginosa* mutators biofilms display increased microcolony diameter sizes. (A) The dynamics of microcolony diameters for Hex1T (filled circles) and Hex1TMS (open circles) strains is shown. Values represent means ± standard error of 24 random chosen CLSM images for each strain and time point. All differences were tested for statistic significance using the ANOVA with repeated measures test (*P*<0.05). (B) Equivalent results were obtained when microcolony diameter sizes were compared between biofilms formed by the wild-type PAO1 (filled circles) and the mutator PAOMS (open circles) strains.

However, the two mutator strains reproducibly developed into micro-colony structures distinctly different from the wild-type strains, as revealed by the microscopic analysis. In order to quantitatively characterize the micro-colonies formed by each strain, the diameters of single micro-colonies were measured from 24 randomly chosen CLSM micrographs of each strain at 24, 48, 96, and 144 h using LSM Image Browser software. As shown in Figure 2, the diameter of the micro-colonies increased over time for both wild-types and mutators (ANOVA with repeated measures, *P*<0.05). After 24 h of growth the micro-colony diameters were very similar in mutator and wild-type biofilms, but during the next 24 h micro-colony sizes increased faster in the mutator biofilms (Figure 2). Thus, at 48 h micro-colony diameters were 62.4±2.5 µm (95% confidence interval 57.3-67.5) and 35.7±2.5 µm (95% confidence interval 30.7–40.8) for Hex1TMS and Hex1T, respectively. These differences were maintained during the following 96 and 144 h of growth (Figure 2 A). Interestingly, at 48 h Hex1TMS biofilms showed a greater variability in micro-colony diameters (intervals 41.9–115.4 µm) with oversized micro-colonies, which resulted in a higher heterogeneity in biofilm structure. Equivalent results were obtained with the PAO1 strain (Figure 2 B), indicating that mutator biofilms develop with accelerated growth compared to their wild-type counterparts, reaching maturity states at earlier time points.

Furthermore, in mutator biofilms central voids in micro-colonies were frequently observed already after 96 h of incubation, whereas in non-mutator biofilms these structures were hardly detected during the 6 days of biofilm growth tested (Figure S2 A and B). Previous reports have documented the presence of large voids in micro-colonies after 7 days of growth [56], and it has been proposed that a “threshold diameter” of approximately 80 µm is required for the onset of the seeding dispersal from the interior of the microcolonies [57]. Consistent with this, the stage where mutators micro-colonies reached this threshold diameter [71.5±3.4 and 84.3±3.1 µm micro-colony diameters (mean ± SE) for Hex1TMS at 96 h and 144 h, respectively], was the same at which the frequency of hollow micro-colonies was increased (Figure S2 B), indicating that the greater rate of dispersal could be associated with the accelerated development of mutator biofilms.

### Increased phenotypic diversification in mutator biofilms

**Figure 3:**
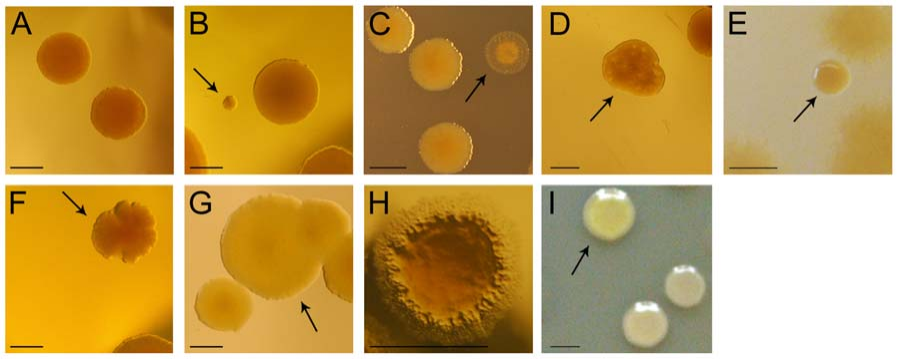
Hypermutability due to *mutS* deficiency increases morphotypic diversification in continuous flow-cell biofilms. Colony morphologies of morphotypic variants isolated from *P. aeruginosa* mutator bioflms. (A) Normal; (B) Small; (C) Translucent; (D) Wrinkled; (E) Mucoid-like; (F) Undulate; (G) Large; (H) Filiform; (I) Hyper-pigmented. Bars indicate 1 cm.

**Figure 4:**
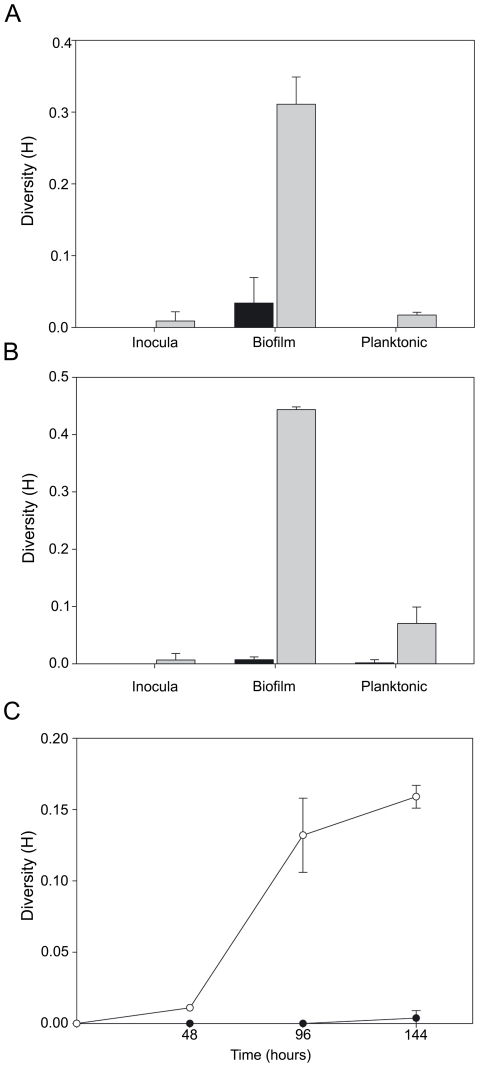
Diversity shown by *P. aeruginosa* mutator strains is intrinsic of flow-cell biofilm mode of growth. The diversity index Shannon-Weaver H was calculated for non-mutator strains Hex1T (A, black bars) and PAO1 (B, black bars) and for mutator strains Hex1TMS (A, gray bars) and PAOMS (B, gray bars) in the inocula from which biofilms were initiated; in 6-day-old flow-chambers biofilms and in planktonic subcultures grown for 6 days with constant agitation. (C) Diversity was also analyzed in the effluents from Hex1T (filled circles) and Hex1TMS biofilms (open circles) at 48, 96 and 144 h of biofilm growth. All values represent means ± SD of three experiments performed by triplicate. The differences were analyzed for statistical significance using the Student *t* test.

Biofilms often develop into spatially and temporally heterogeneous 3D-structures [35], which may provide multiple ecological niches selecting for phenotypically distinct subpopulations [58]. In addition to niche-associated modifications in gene expression (cell physiology), the acquisition of mutations and chromosomal rearrangements are thought to play important roles in the generation of biofilm-adapted phenotypes [35], [51]. Consistently, several reports have previously described that *in vitro P. aeruginosa* biofilms undergo extensive genetic diversification [30], [44], [46], [47], [51]. Moreover, inactivation of MRS was shown to increase phenotypic diversity resulting from phase variation in *Neisseria meningitidis* [59] and mutation-driven phenotypic switches in *P. aeruginosa* [6]-[8], thus increasing adaptation and facilitating niche expansion. Based on these antecedents, we examined the impact of MRS deficiency on phenotypic diversification of *P. aeruginosa* biofilms. For this purpose, 6-day-old biofilms of Hex1T, Hex1TMS, PAO1 and PAOMS were harvested from flow-cells and plated on LB-agar. After 48 h of incubation approximately 10^4^ colonies from each strain were visually inspected for colony morphology variation. As shown in Table 1, variation in colony morphology was greatly increased in both Hex1TMS and PAOMS mutator biofilms. Approximately 20-35% of the total CFUs were different from the standard parental morphotype, and at least 8 distinct morphotypes could be recognized. Among them, flat colonies with translucent edges (T), wrinkled colonies (W) as well as large (L) and smooth small (S) colony variants were the most abundant types (Table 1, Figure 3). These morphotypes were likely consequences of genetic changes, since reversion was not observed after at least two passages on LB-agar plates. Morphotypical diversity was also observed in both non-mutator wild-type strains, but to a much lower extent, as evidenced not only by the smaller number of morphotypes detected, but also by their significantly lower frequency (Table 1, Figure 4 A, Student *t* test *P<0.05*).

**Table 1:** Frequency of occurrence of morphotypic variants from 144 h-old biofilms of Hex1T, Hex1TMS, PAO1 and PAOMS.

	Variants (%)								
	Normal (N)	Small (S)	Translucent (T)	Wrinkled (W)	Mucoid (M)	Large (L)	Undulate (U)	Filamentous (F)	Hyper-pigmentated (H)
Hex1T	98.48	0.02	1.48	0	0.01	0	0.01	0	0
Hex1TMS	77.88	0.59	16.75	3.12	0.04	1.40	0.12	0.08	0.02
PAO1	99.77	0.13	0.10	0	0	0	0	0	0
PAOMS	63.92	5.45	14.95	0.19	0.19	15	0.30	0	0

Values are expressed as percentage of the 10^4^ CFU screened.

Differences in morphotypical diversification between mutators and non-mutators were mainly observed during biofilm growth, since colony diversity for all strains was very low in the biofilm inocula as well as in experiments of planktonic growth conducted in homogeneous aerated liquid media (Figure 4 A and B). Increasing diversity was also observed when biofilm effluents from Hex1TMS were analyzed at 48, 96 and 144 h post-inoculation, respectively (Figure 4 C). In fact, whereas effluents obtained from the Hex1T non-mutator strain showed a poorly detectable diversity throughout the entire time period of biofilm development, a progressive morphological diversification was apparent from mutator effluents reaching a maximum at 144 h (Figure 4 C).

It is important to note that the most abundant variants (T, S, W and L) obtained from mutator biofilms were distinguishable from their respective ancestors not only by their colony morphologies but also by properties such as motility, exopolysacharide matrix and iron uptake capability (Figure S3 and Table S1). This clearly suggests that colony morphology variation is a useful indicator of population diversification as was shown previously in several publications by Rainey *et al*. [36], [60] and Hansen *et al.* [42], [61].

Although previous genetic studies have proposed possible molecular bases underlying the emergence of *P. aeruginosa* biofilm-related colony morphologies [46], [62]–[64], mutations that generate each colony type observed in this study remains to be elucidated.

### Competitive advantage of mutators in biofilm growth

**Figure 5:**
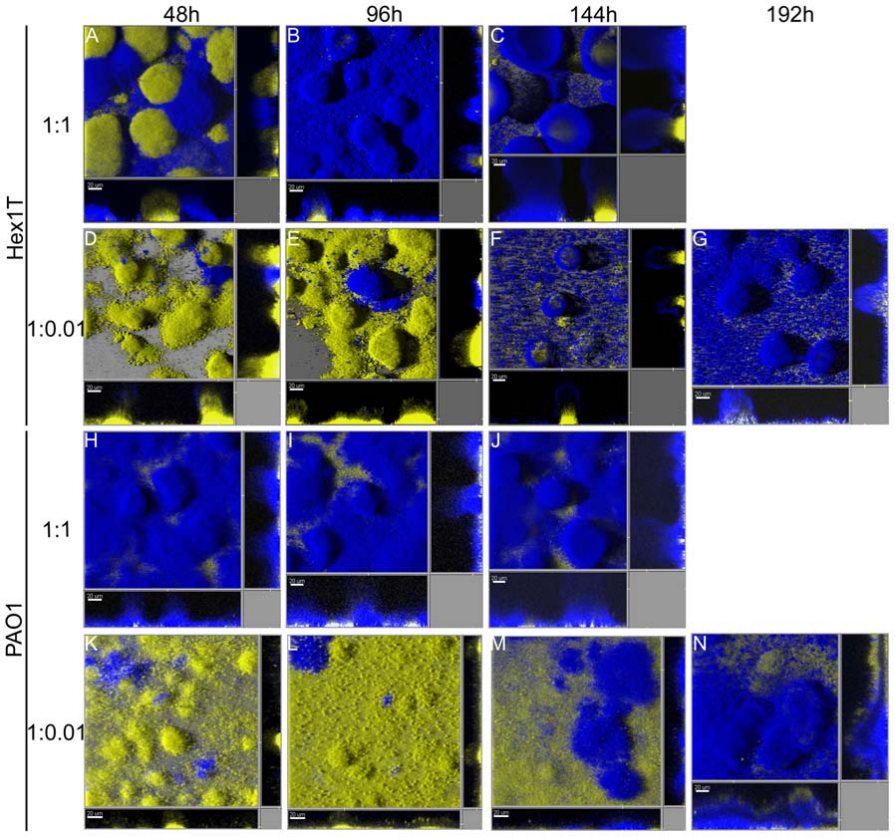
*P. aeruginosa* mutator strains show a greater adaptability to biofilm growth in flow chambers. Biofilm competition experiments between Hex1T:Hex1TMS (A-G) and PAO1:PAOMS (H-N) were initiated with a 1∶1 and 1∶0.01 mixtures of yellow fluorescent non-mutator and cyan fluorescent mutator bacteria and grown in FAB glucose media. The structural development in the biofilm was followed by CLSM for 144 and 196 h for 1∶1 and 1∶0.01 competition ratios, respectively. The images shown are representative 3D-IMARIS shadow projection micrographs.

The results presented above show that *P. aeruginosa* MRS-deficient mutators exhibit greater micro-colony development (Figure 2) with enhanced morphotypical diversification in flow-chamber biofilm populations (Table 1 and Figures 3 and 4). One possible explanation is that mutators may provide the biofilm community with a larger proportion of adapted mutants, which can be enriched by selection for better growth in the biofilm environment. It is therefore possible that mutator strains have a competitive advantage over wild-type populations during biofilm development. In an attempt to probe this possibility and to investigate the role of hypermutability on the evolution of *P. aeruginosa* biofilms, we performed competition experiments between mutator and wild-type strains. For this purpose, flow-cell chambers were co-inoculated with a 1∶1 initial ratio of differentially YFP-tagged wild-type and CFP-tagged mutator cells, and the spatial distribution of the bacteria was recorded as the biofilm developed (Figure 5). After 48 h of incubation, separated yellow and blue micro-colonies were observed in the mixed biofilms (Figure 5), revealing a clonal origin of micro-colonies as previously documented [32]. However, in 4-day-old biofilms the mutators became dominant occupying almost the entire biofilm at day 6 (Figure 5 B, C and I, J). At this time, the biofilms were harvested and plated on LB-agar in order to determine the actual sub-population sizes. Consistent with the microscopic observations the mutator cells outnumbered the wild-type cells by a factor of 5.5 for Hex1T and 6.3 for PAO1. In addition, planktonic growth competition assays were performed in liquid aerated media by co-inoculating with 1∶1 initial ratio of wild-type and mutator strains. Interestingly, Hex1TMS:Hex1T and PAOMS:PAO1 final ratios of 0.11 and 0.56, respectively, were obtained after six days of growth in FAB media, showing that the increased competiveness of the mutators was specific for the biofilm mode of growth.

Based on this observation, we next investigated if the mutator biofilm advantage was also observed in asymmetric competition experiments, where their initial proportion was significantly lower than those of wild-types. For this purpose, mixed biofilms with a 1∶0.01 initial ratio of wild-type and *mutS* mutant strains were investigated. As shown in Figure 5, large clusters of wild-type cells still comprised the majority of the biofilms after 96 h of incubation, but after that time point, mutators became more and more dominant, clearly outcompeting the wild-type at 192 h (Figure 5 D-G and K-N), confirming the mutator advantage over wild-type when growing as biofilms.

It is important to note that generation times in liquid media of the Hex1TMS and PAOMS mutants and their wild-type counterparts were identical (not shown), and opposite fluorescence tagging combinations produced the same results (not shown).

### Evidence for adaptive evolution in mutator biofilms

**Figure 6:**
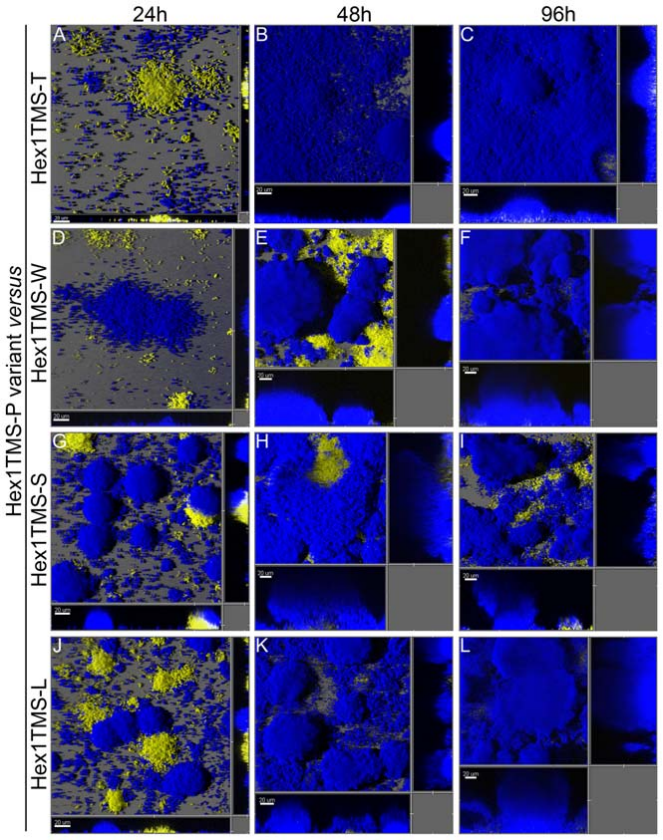
Mutator variants are widely adapted to growth in biofilm flow chambers. Biofilm competition experiments were performed between the parental YFP-tagged Hex1TMS-P strain and the four CFP-tagged mutator variants: translucent (Hex1TMS-T, A-C), wrinkled (Hex1TMS-W, D-F), small (Hex1TMS-S, G-I) and large (Hex1TMS-L, J-L). All mixed biofilms were initiated with a 1∶1 mixture of corresponding bacteria and biofilm development was followed by short periods (96 h) by CLSM. Shown are representative 3D-IMARIS shadow projection micrographs.

In order to examine whether the increased competiveness of the mutator strains in biofilms is related to their increased rates of diversification, we chose the most abundant morpho-variants (T, W, S and L now named Hex1TMS-T, W, S and L respectively) obtained from Hex1TMS biofilms, and investigated their competiveness in biofilms inoculated with a 1∶1 ratio of each of these CFP- tagged variants and the YFP-tagged Hex1TMS parental clone (now named Hex1TMS-P). At 24 h of incubation the variant Hex1TMS-T was present as isolated and small clusters of cells, and the number of Hex1TMS-P and Hex1TMS-T cells was roughly the same (Figure 6 A). In contrast, at the same stage of biofilm development the variants Hex1TMS-W, S and L showed significantly enhanced micro-colony development with cell clusters that reached a more advanced maturation level (Figure 6 D, G and J). At 48 h all variants tested outcompeted the Hex1TMS-P strain and constituted the majority of the biofilms after 96 h of incubation (Figure 6). Importantly, biofilms formed by each variant displayed a particular architecture that was structurally different from that of Hex1TMS-P: (i) Hex1TMS-T formed thinner biofilms with micro-colonies that mostly did not reach 20 µm in height, but which covered the whole surface with a compact carpet of cells (Figure 6 C); (ii) Hex1TMS-S formed typical mature mushroom-shaped micro-colonies more than 20 µm in height although biofilms displayed a poorer surface area coverage (Figure 6 I); (iii) Hex1TMS-L and Hex1TMS-W formed hyper biofilms with the tallest mushroom-shaped structures (Figure 6 F and L).

These results suggest that the distinct morphotypes tested here may have specialized biofilm functions, which provide them with competitive advantages during biofilm development.

## Discussion

In this communication we have investigated the combined roles of biofilm growth and increased mutations rates with respect to a possible reproduction of some of the adaptive processes and outcomes in CF airways, when performed in *in vitro* flow-cell biofilm set-ups. One of the interesting questions is whether biofilm development and occurrence of mutator variants are somehow linked, and if the two create especially favorable conditions for the establishment of chronic infections based on highly adapted variants. The presented observations of *P. aeruginosa* flow-cells biofilms were then directed towards a clarification of the relationship between the biofilm life-style and the consequences of increased mutation rates in bacterial biofilm populations. The impacts of increased mutation rates, caused by knock-out mutations in the MRS associated gene, *mutS*, were analyzed for two different strains of *P. aeruginosa*. Once micro-colonies had been established, mutator biofilms showed an accelerated and enhanced growth, and developed some micro-colonies with larger diameters producing a structural heterogeneity of the entire biofilm (Figure 2). Micro-colonies constitute the basic structural unit in many sessile communities, and as discrete foci of proliferation they may also represent important sites for mutation and evolution within biofilms [50]. Previous studies report that biofilm communities of *P. aeruginosa* show up to 100-fold increased mutation rates compared to planktonic cultures [52], and that such high rates are associated particularly with cells localized in micro-colonies [50]. Our results support these observations, demonstrating that high mutation rates lead to larger and more heterogeneous micro-colony structures, underlining the importance of mutations and genetic rearrangements as determinants of micro-colony based growth and ultimately biofilm architecture.

In many studied cases biofilm growth has been shown to result in phenotypic diversification for several bacterial species including *P. aeruginosa* [30], [44], [46], [47], [51], [63]. Here, we show that increased mutation rates caused by MRS deficiency further resulted in increased diversification in *P. aeruginosa* mutator biofilms in comparison to isogenic wild-type biofilms (Table 1, Figures 3 and 4). Generation of genetic diversity in biofilms communities has been associated with the seeding dispersal process [57], an active selective cell lysis mechanism that would confer a population survival advantage during further colonization. It was found here that the rates of loss of biomass from the central parts of micro-colonies in mutator biofilms were also increased in comparison with their non-mutator counterparts (Figure S2 B), observations that may be directly related to the higher degree of phenotypic variation in mutator biofilms. Therefore, our results strongly suggest a role for mutagenesis in determining micro-colony growth and biofilm architecture, but also in favoring seeding dispersal and phenotypic diversification.

A central finding from our work is that *P. aeruginosa* MRS-deficient mutator strains effectively outcompeted wild-type bacteria in mixed biofilms (Figure 5) but not in planktonic growth. In *E. coli* populations undergoing adaptation, mutators spontaneously occurring as a consequence of mutations in DNA repair genes can be enriched and become dominant in the population through their co-selection with advantageous mutations (hitch-hiking) [3], [65], [66]. Moreover, it has been experimentally proven that overcoming successive selection barriers results in a great amplification of mutator cells in *E. coli* populations [65], [67] and that, when facing new environments or stressful conditions, mutator phenotypes can speed up adaptation of a bacterial population through increased generation of useful variants [68]. Such is the case of antibiotic resistant variants, shown to drive the amplification of mutator populations under antibiotic exposure in several models, recently including biofilm growth [69]. However, in this work we had the possibility to follow *in situ* how the adaptive advantage of mutator cells is associated with greater rates of diversification during biofilm development. It is important to note that although the experiments reported here were short-term they were carried out in the absence of any exogenous selective pressure (such as addition of antibiotics to the culture media), and that in homogenous planktonic growth competitions experiments the *mutS* mutator strains were not favored. Thus, our results suggest that the specific selective forces governing growth in the heterogeneous biofilm environment drive the competitive advantage of *P. aeruginosa* mutator clones. The observation that distinct and different variants isolated from mutator biofilms (Figure 3; Figure S3) were able to outcompete the mutator parental strain in a second round of flow-cell biofilm cultures (Figure 6) suggests that these variants may have acquired different adaptive mutations that provide a competitive advantage to the biofilm mode of growth. Thus, mutator advantage over wild type when grown in biofilms appears to be associated with greater rate of diversification.

It has been shown that in *P. aeruginosa* biofilms the seeding dispersal [56] and phenotypic variation can be induced by oxidative stress [51], evidenced by the fact that phenotypic diversification in biofilms was inhibited by the addition of antioxidant compounds [51]. In accordance with this, it has also been reported that synthesis of antioxidant enzymes is down-regulated in *P. aeruginosa* biofilms, which may enhance the occurrence of mutagenic events due to accumulation of DNA oxidative damage events [52]. It has been further shown that oxidative stress induced mutagenesis most likely is based on RecA dependent recombination repair mechanisms, but not on MRS or Pol IV error prone DNA polymerase [51]. Thus, even in biofilms consisting of only wild-type cells, oxidative stress-induced mutagenesis can lead to diversification of the biofilm population. However, for *P. aeruginosa* MRS-deficient strains, the mutagenic mechanisms involved in biofilm-associated phenotypic diversification may not be exclusively related to oxidative stress, but to alternative pathways. This observation would imply that in MRS-deficient cells the evolutionary mechanisms allowing adaptation to a sessile environment not only produce a comparatively enhanced (quantitative) adaptation, but also use distinct (qualitative) mechanistic pathways. In fact, based on the postreplicative action and mismatch affinity of MutS, MRS deficiency may determine an increased mutability biased towards a specific spectrum of mutations [8], or only at some loci [9]. Moreover, mechanisms that lead to an increased genetic/phenotypic variation could well include phase variation, adaptive mutations, genetic rearrangements, enhanced transfer through conjugation, and transformation mechanisms which have been documented to be activated in MRS deficient cells [7], [8], [10], [59], [70], [71]

It is likely that these mutagenic processes also take place in CF airways, exposing the *P. aeruginosa* population to mutagenic factors no matter if the bacteria live as planktonic cells in the mucus, as biofilms encased in alginate or in any other state, and the increased mutation rates most likely offer excellent possibilities for accumulation of fitness increasing mutations if the population size is big enough. Translating the present results obtained from flow-cell biofilms into relevant suggestions for the adaptive processes which *P. aeruginosa* undergoes during its infection cycle in CF patient airways, we can now hypothesize that the occurrence of mutator cells in the *P. aeruginosa* airway biofilm population may lead to a subsequent dominance of the mutators due to the efficient biofilm associated out-competition of non-mutators. The consequence is population diversification as it has been observed for many CF patients. Variants with increased capacity to form biofilm in the airway environment will be expected to appear in the population. This hypothesis explains the links between three important aspects of *P. aeruginosa* adaptation in CF airways: the biofilm state of growth, the fixation and dominance in many patients of mutators, and the generation of diversity in the bacterial population.

## Materials and Methods

### Bacterial strains and growth conditions

*P. aeruginosa* reference strain PAO1 [53] and the environmental strain Hex1T [54], originally isolated from hydrocarbon-contaminated soil, were used. Isogenic PAO1 and Hex1T *mutS* mutants [6], [55] were used in the present study and referred as PAOMS and Hex1TMS, respectively. Strains were stored at −70°C in 30% (v/v) glycerol and subcultured from storage onto Luria Bertani (LB) medium. *P. aeruginosa* PAO1 strains were fluorescently tagged at the *att* intergenic neutral chromosomal locus with *gfp*, *cfp* or *yfp* with miniTn*7* constructs as previously described [31]. Hex1T strains were electroporated with pJBA128 [72], pJB1197 and pJB1199 plasmids carrying GFP, YFP and CGP fluorescent proteins, respectively. Modified FAB medium [73] supplemented with 0.3 mM glucose was used for biofilm cultivation. Biofilms and batch cultures were grown at 30°C. Antibiotics were used at the following concentrations: 30 µg/ml gentamicin (Gm); 250 µg/ml kanamycin (Km); 200 µg/ml streptomycin (Sm); 60 µg/ml tetracicline.

### Flow-cell biofilm experiments

Biofilms were grown at 30°C in flow chambers with individual channel dimensions of 1×4×40 mm. The flow system was assembled and prepared as described previously [74]. Each biofilm experiment was conducted by using frozen cells as the starting stock. In order to ensure that attachment mediated by type IV pili and subsequent structured biofilm formation were not affected [31], [75], before each biofilm culture, twitching motility assays were routinely performed as previously described [43]. A twitching positive colony from a plate was used to inoculate test tubes containing LB media and grown at 30°C for 16 h with aeration. Cultures were diluted to an OD_600_ of 0.001 in 0.9% NaCl and used for inoculation; 250 µl were injected with a small syringe. After inoculation flow channels were left without flow for 1 h to allow bacterial adherence, after which medium flow (0.2 mm/s) was started using a Watson Marlow 205S peristaltic pump.

For the analysis of the structure and evolution of *P. aeruginosa* PAO1, Hex1T, PAOMS and Hex1TMS biofilms, three independent biofilm experiments were performed. In every round, each strain was grown by triplicate, running three separate channels, and from each channel four image stacks were acquired at different time point (15, 24, 48, 96 and 144 h after inoculation). Thus, 36 image stacks were analyzed for each strain at each time point. In all the experiments, images were acquired from random positions in the upper part of the flow channel, at a distance of 5–10 mm from the inlet.

### Microscopy and image analysis

All microscopic observations were performed on a Zeiss LSM510 confocal laser scanning microscope (CLSM) (Carl Zeiss, Jena, Germany) equipped with an argon laser, detector and filter sets for monitoring of *gfp*, *cfp* and *yfp* expression. Images were obtained by using a 40×/1.3 Plan-Neofluar oil objective and were processed using the COMSTAT image analysis program [76]. Simulated three-dimensional images and sections were generated by using the IMARIS software package (Bitplane AG, Zurich, Switzerland).

### Phenotypic diversification experiments

#### i. Biofilms

To study phenotypic diversification, at 144 h of growth biofilms were harvested and adequate dilutions were plated on LB agar and incubated for 48 h at 37°C. Morphology of approximately 10^4^ colonies per strain from three independent experiments was examined by visual inspection.

Diversity from the biofilm effluents was monitored at 48, 96 and 144 h, by collecting into sterile tubes 1 ml of effluent media of *P. aeruginosa* Hex1T and Hex1TMS strains. Serial dilutions of each effluent were plated on LB agar and examined for morphotypic colony variants (at least 10^4^ colonies). Morphotypic colony diversification in the inocula was tested by direct plating of part of bacterial cultures used to establish biofilms.

#### ii. Liquid culture. To test

morphotypic colony diversification in planktonic cultures, bacteria (10^7^ cells/ml) were incubated aerobically in FAB medium (10 ml, 30 mM glucose) at 30°C for 24 h, diluted 1/100 into fresh FAB and incubated again for 24 h. This cycle was repeated for four times. On sixth day, serial dilutions were plated on LB agar and incubated at 37°C for 48 h. Morphology of approximately 10^4^ colonies per strain from three independent experiments was examined by visual inspection.

### Diversity

Diversity (*H*) was calculated using the Shannon-Weaver index [*H* = (*N* log*N*−Σ*n* log *n_i_*)/*N*], where *N* is the total number of individuals and *n_i_* is the number of individuals of each phenotype.

### Competition experiments

#### i. Biofilms

Pairs of PAO1:PAOMS and Hex1T:Hex1TMS strains differentially tagged with yellow (wild types) and cyan (mutators) fluorescent proteins were co-cultured in flow cells and dynamics of mixed biofilms was followed by CLSM. Initial ratios were 1∶1 and 1∶0.01 and final ratios for 1∶1 competitions were tested at 144 h by harvesting biofilms and plating serial dilutions on LB agar either containing 200 µg/ml of streptomycin or 30 µg/ml of gentamicin for selection PAO1 and PAOMS, respectively, or containing 250 µg/ml of kanamycin for Hex1TMS selection. Triplicate channels of each mixed culture were examined in at least two independent experiments with an additional run with the opposite color combination.

For competition experiments between colony morphology variants and the ancestor Hex1TMS-P strain the four most prevalent phenotypes (S, L, W and T), directly obtained from the diversification analysis were resuspended in NaCl 0.9% and inoculated in the mixed biofilm. Initial ratios were 1∶1 ratio and each colony morphology variant was tagged with the *cfp* gene differently from the YFP-tagged Hex1TMS ancestor strain. Triplicate channels of each strain pair were run in at least two independent experiments.

#### ii. Liquid cultures

For competition assays in liquid aerated media, pairs of PAO1:PAOMS and Hex1T:Hex1TMS strains were co-cultured in 10 ml of FAB media supplemented with 30 µM glucose at a initial 1∶1 ratio (approximately 10^7^ cells/ml) and incubated at 30°C for 24 h. Then 100 µl of a 10^−4^ dilution of these cultures were inoculated into fresh FAB and incubated again for 24 h. This cycle was repeated four additional times and on the sixth day, serial dilutions were plated on LB agar containing 200 µg/ml of streptomycin and LB-agar containing 30 µg/ml of gentamicin for selection PAO1 and PAOMS, respectively, or containing 250 µg/ml of kanamycin for Hex1TMS selection. The plates were incubated at 37°C for 24 h. The experiment was repeated four times.

### Statistical analysis

Structural data were compared using a Student *t* test. In all cases, *P* values of <0.05 were considered statistically significant. Analysis of variance [94] with repeated measures was used to compare the differences in microcolony diameters assessing the interaction between time (24, 48, 96 and 144 h) and strains using SPSS Statistic Standard 15.0 software (IBM). Logarithmic transformations were applied for all the data so that they met with normal distribution and homogeneity of the variances. All comparisons were determined after Bonferroni's correction of *P* values.

## Supporting Information

Figure S1 **Biofilm parameters analyzed by using COMSTAT software.** Biomass, maximum thickness and surface-to-volume ratio were calculated at 15, 24, 48, 96 and 144 h after inoculation in flow-cell biofilms formed by Hex1T and PAO1 strains and their respective isogenic Hex1TMS and PAOMS strains. COMSTAT was carried out from images acquired from random positions in the inner part of the flow channel. The asterisks indicate statistical differences (*P<0.05*) between mutator (white bars) and wild-type (black bars) strains. Results represent the mean ± SEM of three independent experiments.
(TIF)

Figure S2 **Seeding dispersal is highly frequent in** ***P. aeruginosa*** **Hex1TMS biofilms.** (A) Confocal micrograph showing a *P. aeruginosa* Hex1TMS hollow microcolony. The image was obtained 96 h after flow-cells were inoculated. Subpopulation of swimming cells inside the microcolony can be visualized. (B) Number of hollow microcolonies/cm^2^ in biofilms formed by Hex1T (black bars) and Hex1TMS (grey bars). Quantification was performed by assessing the number of void microcolonies in 16 mm^2^ flow-channel area for each strain at 48, 96 and 144 h of biofilm incubation. The results are representative of measurements of three experiments.
(TIF)

Figure S3 **Characterization of colony morphology of** ***P. aeruginosa*** **He1TMS variants obtained from mutator biofilms.** Overnight cultures (5 µl) of bacterial cultures were spot on LB-agar plates supplemented with 40 µg/ml of Congo red dye (A) or 4 mM FeSO_4_ (B) and incubated at 30°C for 48 h. Hex1T wild-type and the parental Hex1TMS-P mutator strains showed morphotypes typically redish (with concentrically colorless rings), and brownish when plated on Congo red and on high iron media respectively. All Hex1TMS-T, S, W and L morphotypic variants displayed colorless colonies when plated on iron supplemented media. On Congo red, colonies were shiny autolytic for Hex1TMS-T; light pigmented with red edges for Hex1TMS-W; and hyperpigmented for Hex1TMS S and L.
(TIF)

Table S1 Motility assays of *P. aeruginosa* Hex1MS-P parental morphotype and Hex1MS-T, -W, -S and –L morphotypes biofilm variants.
(DOC)
