# Supplementary figures and images for: Evolution and Adaptation in Pseudomonas aeruginosa Biofilms Driven by Mismatch Repair System-Deficient Mutators
Source: PLoS One. 2011 Nov 17;6(11):e27842. doi: 10.1371/journal.pone.0027842 (PMC3219696; doi:10.1371/journal.pone.0027842)

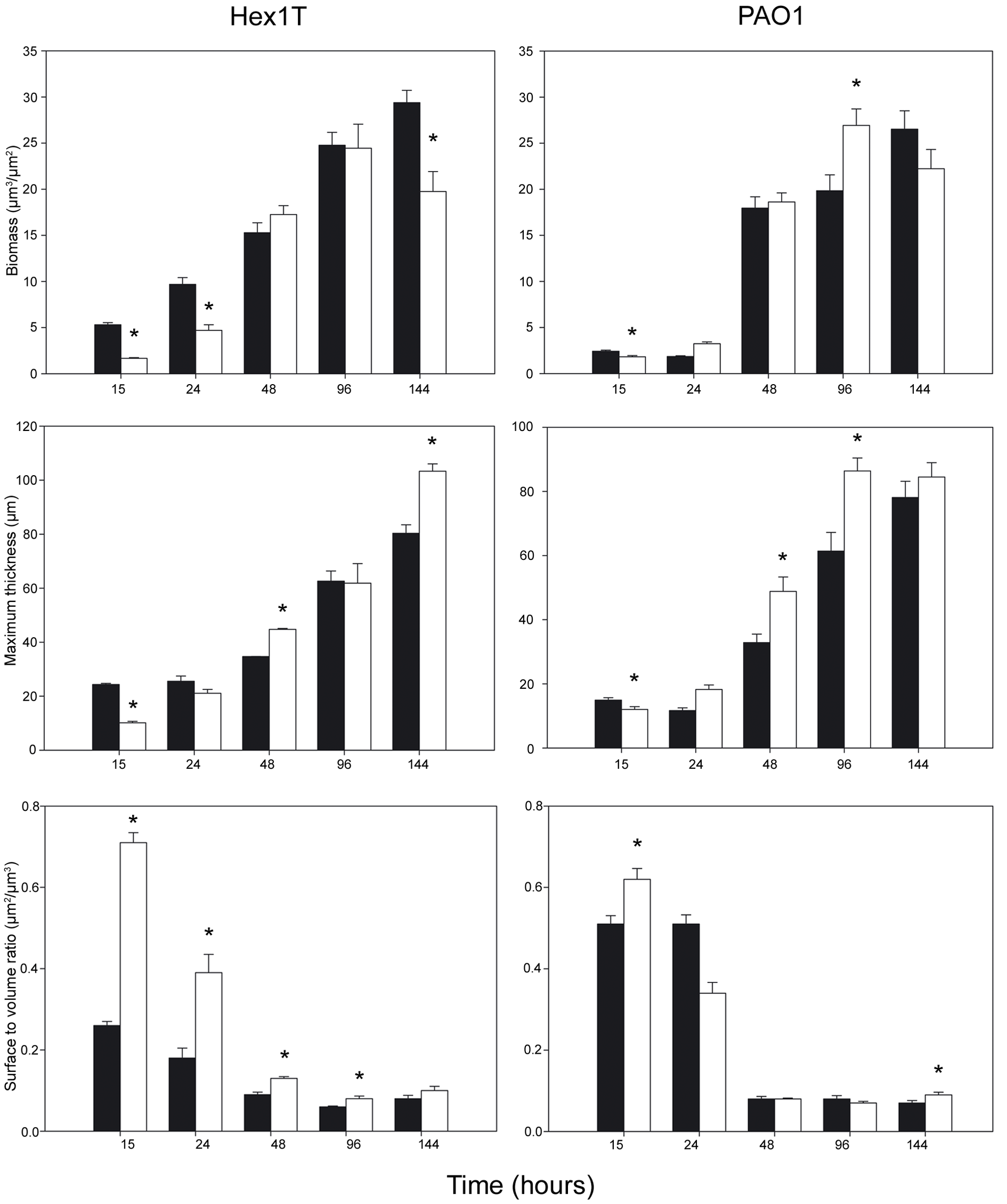

Supplement: Figure S1 — Biofilm parameters analyzed by using COMSTAT software. Biomass, maximum thickness and surface-to-volume ratio were calculated at 15, 24, 48, 96 and 144 h after inoculation in flow-cell biofilms formed by Hex1T and PAO1 strains and their respective isogenic Hex1TMS and PAOMS strains. COMSTAT was carried out from images acquired from random positions in the inner part of the flow channel. The asterisks indicate statistical differences (P<0.05) between mutator (white bars) and wild-type (black bars) strains. Results represent the mean ± SEM of three independent experiments. (TIF) [file pone.0027842.s001.tif]

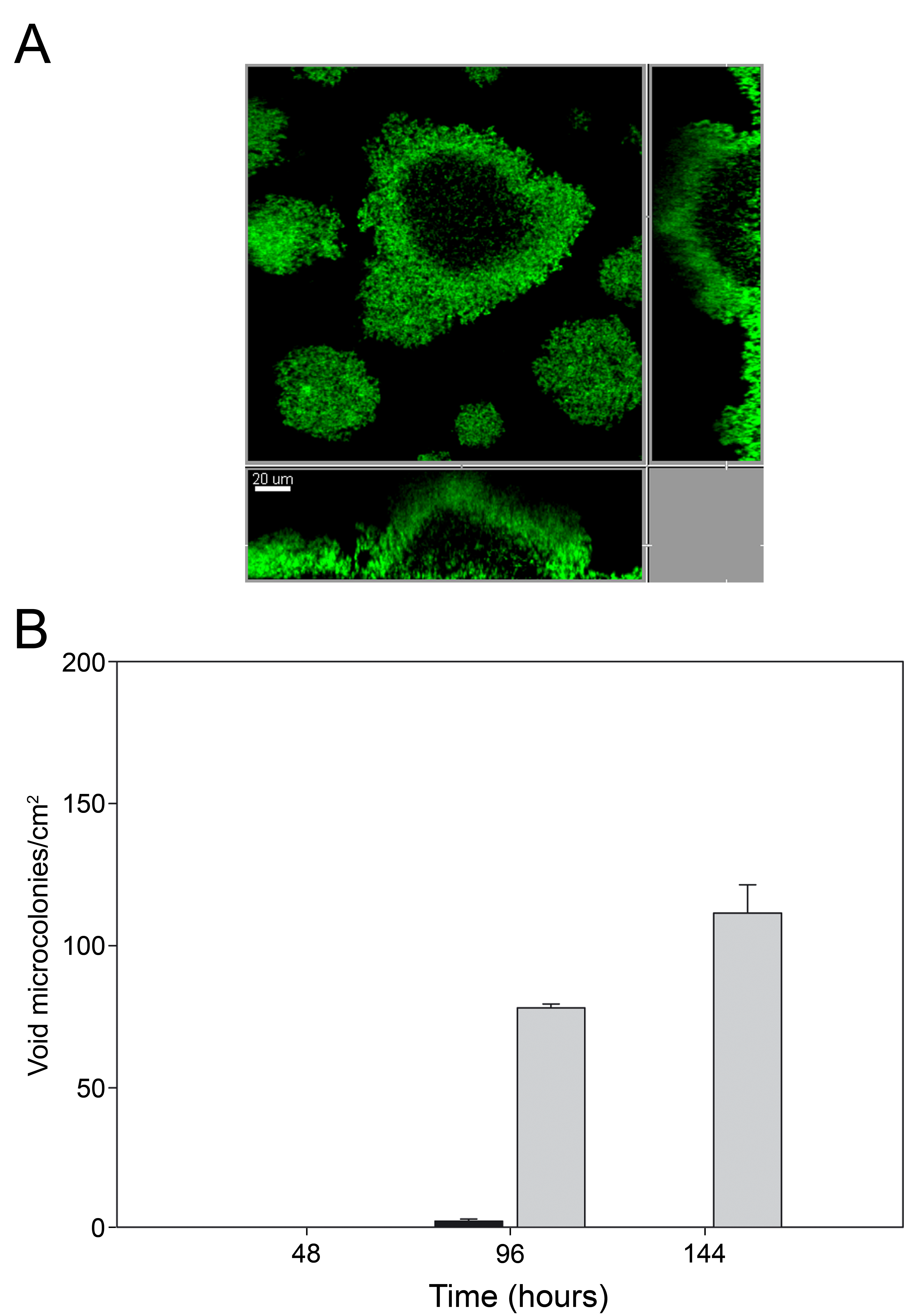

Supplement: Figure S2 — Seeding dispersal is highly frequent in P. aeruginosa Hex1TMS biofilms. (A) Confocal micrograph showing a P. aeruginosa Hex1TMS hollow microcolony. The image was obtained 96 h after flow-cells were inoculated. Subpopulation of swimming cells inside the microcolony can be visualized. (B) Number of hollow microcolonies/cm2 in biofilms formed by Hex1T (black bars) and Hex1TMS (grey bars). Quantification was performed by assessing the number of void microcolonies in 16 mm2 flow-channel area for each strain at 48, 96 and 144 h of biofilm incubation. The results are representative of measurements of three experiments. (TIF) [file pone.0027842.s002.tif]

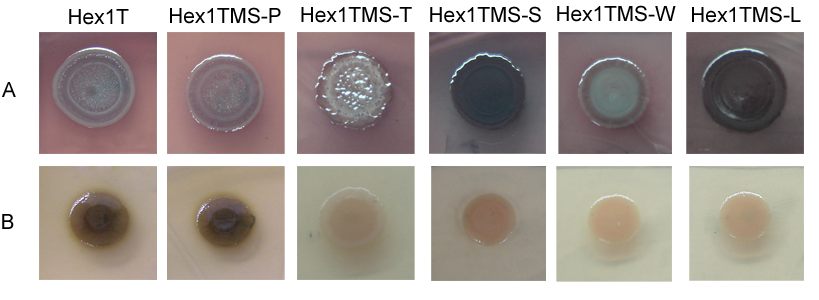

Supplement: Figure S3 — Characterization of colony morphology of P. aeruginosa He1TMS variants obtained from mutator biofilms. Overnight cultures (5 µl) of bacterial cultures were spot on LB-agar plates supplemented with 40 µg/ml of Congo red dye (A) or 4 mM FeSO4 (B) and incubated at 30°C for 48 h. Hex1T wild-type and the parental Hex1TMS-P mutator strains showed morphotypes typically redish (with concentrically colorless rings), and brownish when plated on Congo red and on high iron media respectively. All Hex1TMS-T, S, W and L morphotypic variants displayed colorless colonies when plated on iron supplemented media. On Congo red, colonies were shiny autolytic for Hex1TMS-T; light pigmented with red edges for Hex1TMS-W; and hyperpigmented for Hex1TMS S and L. (TIF) [file pone.0027842.s003.tif]
