# Supplementary material for: Evolution and Adaptation in Pseudomonas aeruginosa Biofilms Driven by Mismatch Repair System-Deficient Mutators
Source: PLoS One. 2011 Nov 17;6(11):e27842. doi: 10.1371/journal.pone.0027842 (PMC3219696; doi:10.1371/journal.pone.0027842)
Supplement: Table S1 — Motility assays of P. aeruginosa Hex1MS-P parental morphotype and Hex1MS-T, -W, -S and –L morphotypes biofilm variants. (DOC) [file pone.0027842.s004.doc]

Table S1: Motility assays of *P. aeruginosa* Hex1MS-P parental morphotype and Hex1MS-T, -W, -S and –L morphotypes biofilm variants.

| Strain | Swarming^a^ | Twitching^b^ | Swiming^c^ |
| --- | --- | --- | --- |
| Hex1TMS-P | 1.07±0.02 | 1.43±0.51 | 6.66±0.58 |
| Hex1TMS-T | 1.00±0.10 | 0.50±0.00* | 3.67±0.29* |
| Hex1TMS-W | 0.90±0.00* | 0.50±0.00* | 5.30±0.50* |
| Hex1TMS-S | 0.73±0.06* | 0.50±0.10* | 5.76±1.54 |
| Hex1TMS-L | 1.10±0.17 | 0.30±0.00* | 6.03±1.08 |

In all cases diameter of motility areas are expressed in cm. ^a^Assayed on 0.5 % agar plate as previously described [44]. ^b^Assayed on 1 % agar plate as previously described [44]. ^c^Assayed on 0.3 % agar plate as previously described [44].

*Indicates values that are statistically different from Hex1TMS-P (*P<*0.05)
